# ADAM17 inhibition enhances platinum efficiency in ovarian cancer

**DOI:** 10.18632/oncotarget.24682

**Published:** 2018-03-23

**Authors:** Nina Hedemann, Christoph Rogmans, Susanne Sebens, Daniela Wesch, Manuel Reichert, Dirk Schmidt-Arras, Hans-Heinrich Oberg, Ulrich Pecks, Marion van Mackelenbergh, Jörg Weimer, Norbert Arnold, Nicolai Maass, Dirk O. Bauerschlag

**Affiliations:** ^1^ Department of Gynecology and Obstetrics, Christian-Albrechts-University Kiel and University Medical Center Schleswig-Holstein Campus Kiel, Kiel, Germany; ^2^ Institute for Experimental Cancer Research, Christian-Albrechts-University Kiel and University Medical Center Schleswig-Holstein Campus Kiel, Kiel, Germany; ^3^ Institute of Immunology, Christian-Albrechts-University and University Medical Center Schleswig-Holstein Campus Kiel, Kiel, Germany; ^4^ Institute of Biochemistry, Christian-Albrechts-University Kiel, Kiel, Germany

**Keywords:** ovarian cancer, chemo resistance, ADAM17, amphiregulin, tumor therapy

## Abstract

Chemotherapeutic resistance evolves in about 70 % of ovarian cancer patients and is a major cause of death in this tumor entity. Novel approaches to overcome these therapeutic limitations are therefore highly warranted. A disintegrin and metalloprotease 17 (ADAM17) is highly expressed in ovarian cancer and required for releasing epidermal growth factor receptor (EGFR) ligands like amphiregulin (AREG). This factor has recently been detected in ascites of advanced stage ovarian cancer patients. However, it is not well understood, whether and how ADAM17 might contribute to chemo resistance of ovarian cancer.

In this study, we identified ADAM17 as an essential upstream regulator of AREG release under chemotherapeutic treatment in ovarian cancer cell lines and patient derived cells. In the majority of ovarian cancer cells cisplatin treatment resulted in enhanced ADAM17 activity, as shown by an increased shedding of AREG. Moreover, both mRNA and the protein content of AREG were dose-dependently increased by cisplatin exposure. Consequently, cisplatin strongly induced phosphorylation of ADAM17-downstream mediators, the EGFR and extracellular signal-regulated kinases (ERK). Phorbol 12-myristate 13-acetate (PMA), similarly to cisplatin, mediated AREG shedding and membrane fading of surface ADAM17.

Inhibition of ADAM17 with either GW280264X or the anti-ADAM17 antibody D1 (A12) as well as silencing of ADAM17 by siRNA selectively reduced AREG release. Thus, ADAM17 inhibition sensitized cancer cells to cisplatin-induced apoptosis, and significantly reduced cell viability.

Based on these findings, we propose that targeting of ADAM17 in parallel to chemotherapeutic treatment suppresses survival pathways and potentially diminish evolving secondary chemo resistance mechanisms.

## INTRODUCTION

Ovarian cancer is the most lethal cancer amongst all gynecological malignancies, with more than 100,000 deaths per year worldwide [1]. This circumstance is mainly due to the late stage diagnosis and fast developing chemotherapeutic resistance. About 2/3 of women do not survive the first five years after diagnosis, mostly in consequence of recurrence [2]. Progress in chemotherapeutic treatment was achieved by introducing the antiangiogenic monoclonal antibody Bevacizumab [3–5]. Even more recently, the concept of synthetic lethality by poly ADP ribose polymerase (PARP)-inhibition by Olaparib and Neraparib [6, 7] gave new hope in treating recurrent ovarian cancer harboring mutations in breast cancer gene 1/2 (BRCA1/2) genes.

Thus, overcoming chemo resistance for decades has been one of the most challenging tasks in successfully treating ovarian cancer [8]. Today, many of these approaches focus on deciphering deregulated signaling pathways in order to reveal novel targets for therapeutic intervention. Previous research covered, among others, the involvement of efflux pumps and survival pathways such as epidermal growth factor receptor (EGFR), mitogen-activated protein kinase (MAPK), phosphoinositid-3 kinase and serine/threonine kinase AKT (PI3K/AKT) [8]. A critical mediator acting upstream of all these survival pathways is a disintegrin and metalloprotease 17 (ADAM17), which is focus of the present study [9–13].

ADAM17 proteolytically cleaves a substantial number of substrates and thus plays an important role in physiologic development and tissue regeneration [14–17]. These substrates include the major activators of the EGFR: epiregulin, transforming growth factor alpha (TGF-alpha), amphiregulin (AREG), and heparin-binding EGF-like growth factor [18]. Overexpression or enhanced activation of ADAM17 in tumor cells has been linked to cancer initiation and progression, mostly *via* EGFR activation [10, 11, 19–21].

For most solid tumors, including lung, gastric, renal, colorectal, pancreatic and ovarian cancer, high expression levels of ADAM17 protein were shown [10, 14, 22, 23]. In breast cancer patients, ADAM17 expression correlates with increased metastatic potential and poor survival rate [24]. Besides, a variety of ADAM17 substrates including the EGFR-ligands AREG and TGF-α were detected in patient-derived ascites of ovarian cancer patients, suggesting that ADAM17 is highly active in these patients [25].

Although recent research elucidated the mechanisms of ADAM17 activation, expression and blocking [10, 26–28], adjuvant inhibition of ADAM17 to chemotherapeutic treatment has not been assessed, yet. Kyula and coworkers recently described that ADAM17 was activated in colorectal cancer cells after 5-fluorouracil (5-FU) treatment [29]. This activation leads to an increased shedding of the EGFR ligands, TGF-alpha and AREG and an enhanced EGFR-phosphorylation. Moreover, overexpression of ADAM17 decreased the effect of chemotherapeutic treatment on tumor growth and apoptosis [29].

As ovarian cancer patients are mostly affected by chemo resistance and recurrent disease, we aimed to elucidate the impact of ADAM17 in this particular tumor entity [2].

Because enhanced EGFR, PI3K and MAPK signaling play an important role in chemo resistance and ADAM17 acts upstream of these pathways, we asked, if chemotherapeutic treatment directly impacts ADAM17 protein expression or activation and how this correlates to the cellular expression and release of the ADAM17 substrate AREG and EGFR activation. Moreover, we investigated whether inhibition of ADAM17 can (re-)sensitize ovarian cancer cells to chemotherapeutic treatment.

This study identified a novel role of ADAM17 in promoting chemo resistance in ovarian cancer and it provides evidence that ADAM17 and related signaling pathways including the EGFR and it´s ligands could function as effective targets for combinatorial therapy approaches of this still devastating disease.

## RESULTS

### Cisplatin treatment increases ADAM17 protein amount and AREG release in ovarian cancer cell lines

To investigate whether chemotherapeutic treatment impacts ADAM17 activity, we determined the protein amounts of ADAM17 and its substrate AREG in ovarian cancer cell lines. AREG was chosen as ADAM17 substrate because it was previously identified as one of the most abundant ADAM17 substrates in advanced ovarian cancer [25]. Consequently, we measured AREG release into cell culture supernatants as a surrogate marker for ADAM17 activity.

To do so, we used three established ovarian cancer cell lines with well-defined characteristics: Igrov-1 cells as a cisplatin-intermediate sensitive, EGFR-expressing cell line, A2780 cells as a cisplatin-sensitive, EGFR-negative cell line and cisplatin-resistant Skov-3 cells, exhibiting EGFR expression.

**Figure 1:**
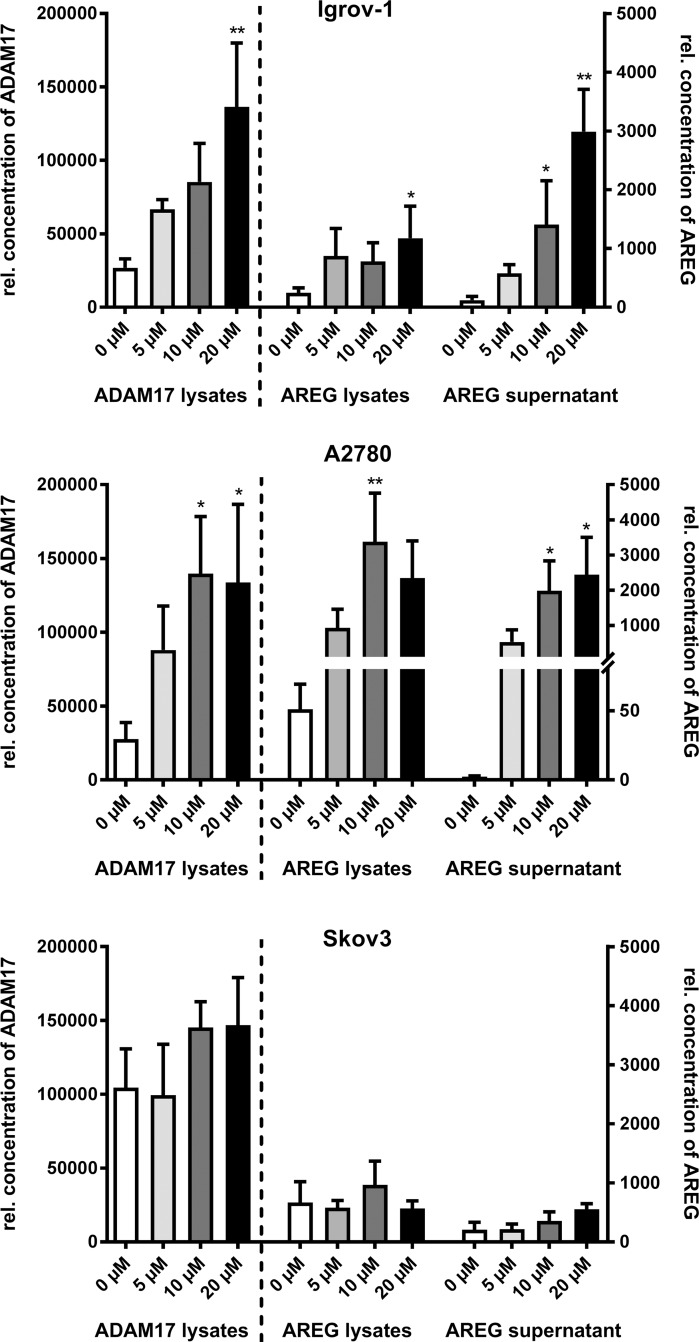
Cisplatin increases ADAM17-dependent AREG release in ovarian carcinoma cell lines After 48 h of cisplatin treatment with the indicated concentrations, cells were trypsinized, counted and lyzed. Optical densities (ODs) of ADAM17 and AREG levels in lysates and AREG amounts in supernatants were measured by sandwich ELISA, and the calculated concentration values were normalized to the total cell number. Cisplatin treatment increased ADAM17-protein amounts (left panel) and AREG levels (center panel) in cell lysates and AREG release into culture supernatants (right panel) in Igrov-1 cells and A2780 cells. Skov-3 cells did not respond to cisplatin treatment. Data from three to five independent experiments per cell line are presented as mean + SEM. Stars indicate significant differences compared to untreated cells (0 μM). Friedman test; P = significance, ^*^ (p<0.05); ^**^ (p<0.01).

An increase in ADAM17 protein levels was observed in cell lysates of Igrov-1 and A2780 cells after cisplatin exposure, using an ADAM17 specific sandwich-ELISA detecting ADAM17, irrespectively of maturation status (p<0.05) (Figure 1 left). By contrast, no elevation in ADAM17 content was found in cisplatin-resistant Skov-3 cells (Figure 1 left). Interestingly, the protein content of ADAM17 was four-fold higher in untreated Skov-3 cells compared to ADAM17 concentration in naïve Igrov-1 and A2780 cells (Figure 1 left). Moreover, we detected the presence of the mature form of ADAM17 (85 kDa) in Igrov-1, A2780 and Skov-3 cells by western blot analysis (Supplementary Figure 1A). In concordance with ELISA results Skov-3 cells present the highest levels of ADAM17, irrespective of cisplatin addition (data not shown, as PCR results were normalized). Cisplatin-dependent induction of DNA-damage was verified by γ-H2Ax (H2A histone family, member X) immunoblotting (Supplementary Figure 1A). However, due to mainly posttranscriptional regulation of ADAM17, mRNA content did not show a significant increase following cisplatin treatment (Supplementary Figure 1B).

Compared to ADAM17 protein amounts there was an even more pronounced, dose-dependent rise in AREG levels upon cisplatin treatment. A five-fold increase was observed in Igrov-1 (p<0.05) and up to 50-fold in A2780 cells (p<0.001), but again not in Skov-3 cells (Figure 1 center). In concordance, mRNA levels of AREG increased by up to eight-fold in Igrov-1 cells and 40-fold in A2780 cells following cisplatin (10 μM) treatment (p<0.05) (Supplementary Figure 1C). AREG expression in Skov-3 cells was only enhanced by four-fold.

We showed a dose-dependent increase of AREG shedding following cisplatin treatment (Figure 1 right) as an indirect measurement of ADAM17 activity. Addition of 20 μM cisplatin enhanced AREG release into supernatants up to 40-fold in Igrov-1 (p<0.001) and A2780 cells (p<0.05) (Figure 1 right). By contrast, Skov-3 cells responded to cisplatin only to a minor extent (Figure 1 right).

Thus, the cisplatin-sensitive cell lines Igrov-1 and A2780 tended to respond to cisplatin treatment by increase of ADAM17 and AREG protein and subsequent AREG release. In contrast to this, the cisplatin-resistant cell line Skov-3 already expressed higher amounts of these proteins irrespective of cisplatin treatment.

### Altered cell surface amounts of ADAM17 reveals preceding activation by cisplatin

**Figure 2:**
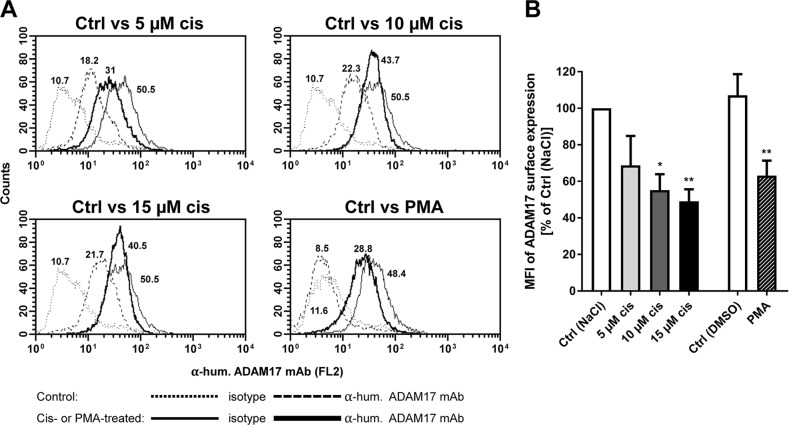
Modulation of ADAM17 on tumor cell surface after cisplatin treatment ADAM17 expression was analyzed on the cell surface of Igrov-1 cells without treatment (control, Ctrl; thin line) or after treatment with the indicated concentrations of Cisplatin (cis) or Phorbolester (PMA) (bold lines) after 48 h. Cells were stained by anti-human TACE (ADAM 17) mAb and by appropriate isotype controls for untreated (Ctrl, dotted line) and treated (dashed lines) samples. Histograms of one representative of five independent experiments are shown **(A)**. The indicated numbers present the mean fluorescence intensity (MFI); shown as the difference of IgG-MFI to ADAM17-MFI. The MFI of control cells [Ctrl (NaCl)] was set to 100 % for comparison of surface ADAM17 expression to cisplatin or PMA treated cells. Mean + SEM of the MFI of five experiments were calculated and are presented as percentage of Ctrl (NaCl). Stars indicate significant differences to the corresponding Ctrl. ANOVA; P = significance, ^*^ (p<0.05); ^**^ (p<0.01) **(B)**.

To answer the question, whether subcellular localization of ADAM17 is modified by cisplatin treatment, FACS analysis of non-permeabilized Igrov-1 cells was performed. Interestingly, surface localization of ADAM17 was reduced dose-dependently in response to cisplatin treatment, by up to 50 percent. Similarly, activation of ADAM17 by Phorbol 12-myristate 13-acetate (PMA), a well-known inducer of ADAM17 activity decreased ADAM17 surface exposure by about 40 percent (p<0.01) (Figure 2).

Thus, cisplatin treatment triggered a similar cellular redistribution of ADAM17 as did ADAM17 activator PMA, being suggestive of a preceding activation process.

### Inhibition of ADAM17 enhances apoptosis and reduces cell viability in cisplatin sensitive cells

**Figure 3:**
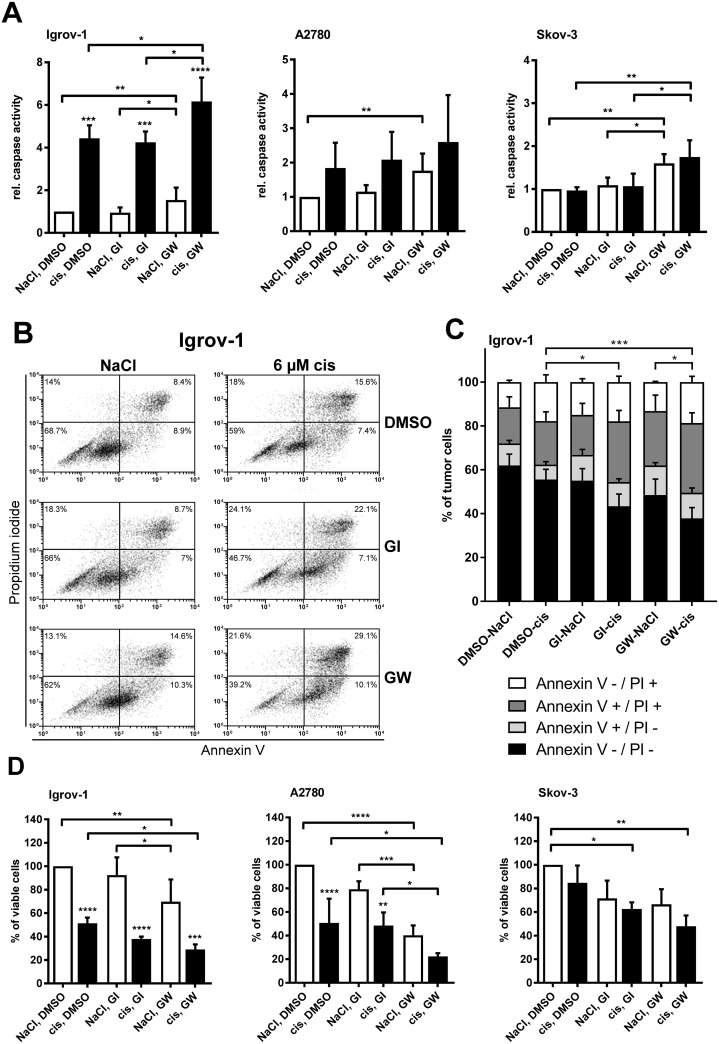
Inhibition of ADAM17 reduces cell viability and enhances apoptosis in cisplatin sensitive cells Cells were treated with 6 μM cisplatin (cis; diluted in NaCl) or NaCl (control) for 24 h for measurement of apoptosis **(A, B, C)** and for 48 h to measure cell viability **(D)**. GI and GW (each diluted in DMSO) were used at a concentration of 3 μM to block ADAM10 and ADAM10/ADAM17, respectively. As a control, cells were treated with DMSO. For comparison of three to five independent experiments the caspase activity of control cells was set to one (A) or the number (no.) of viable cells was set to 100 % of control cells (NaCl and DMSO) (D). Data are presented as mean + SEM. For FACS-analyses, cells were washed and stained with Annexin V-FITC (An V) and Propidium iodide (PI). All analyses were measured on a FACS Calibur (BD Biosciences) (B, C). (B) One representative experiment out of five independent ones is shown with the dot plot analysis and the appropriate percentage of dead cells. (C) The mean distribution + SEM of alive (An V/PI-negative), early apoptotic (An V-positive, PI-negative), late apoptotic/necrotic (An V/PI-positive) or necrotic (An V-negative, PI-positive) tumor cells of five independent experiments are presented. Stars above the black bars indicate significant differences between cisplatin treatment and the corresponding NaCl treatment (left). ANOVA; P = significance, ^*^ (p<0.05); ^**^ (p<0.01); ^***^ (p<0.001); ^****^ (p<0.0001).

Cisplatin treatment increases ADAM17 protein and release of AREG, raising the question whether inhibition of ADAM17 can be used to enhance the effect of chemotherapeutics. Therefore, we assessed the potential of ADAM17 inhibition to increase cytotoxic effects of cisplatin by measuring apoptosis after 24 h treatment. Caspase activation following cisplatin treatment was found to be increased in the cisplatin-sensitive cell lines Igrov-1 (p<0.001) and A2780. Importantly, combined treatment with cisplatin and GW (dual inhibitor of ADAM10 and ADAM17), but not GI (selective ADAM10 inhibitor) increased apoptosis of Igrov-1 cells by up to six-fold compared to untreated cells (p<0.0001) (Figure 3A). This impact was most pronounced in Igrov-1 cells. A2780 cells and Skov-3 cells also demonstrated caspase activation upon ADAM17 blockage (p<0.001) (Figure 3A), but to a lower extent. Interestingly, the cisplatin-resistant cell line Skov-3 showed a pronounced increase in apoptosis by treatment with GW alone being slightly increased by the drug combination (Figure 3A). FACS analyses of Igrov-1 cells (Figure 3B, 3C) using Annexin V (An V)/PI staining confirmed increased apoptosis induction when cisplatin was combined with GW [~20 % enhanced cell death] (p=0.0007) (Figure 3B, 3C). Simultaneous treatment of GI and cisplatin showed similar results but to a minor extent. Inhibition by GW alone already significantly triggered apoptosis compared to control treatment (NaCl, DMSO) by about 12 % (p=0.023), suggesting a critical role of ADAM17 in survival of ovarian cancer cells. Cell viability after a treatment interval of 48 h added up to these results verifying the aforementioned findings. For these experiments, cells were treated either with a dual inhibitor GW (ADAM10 / ADAM17 inhibition) or the selective inhibitor GI (ADAM10 inhibition). We found an increased decline of cell viability by an additional 20 % in Igrov-1 cells (p<0.01) and 30 % in A2780 cells (p<0.01) when compared to cisplatin monotherapy, respectively (Figure 3 D). Applying the inhibitor GW alone, reduced the number of viable cells by 30 % in Igrov-1 (p<0.01) and 60 % in A2780 cells (p<0.0001) (white bars). We did not observe this reduction using the ADAM10 specific inhibitor GI. Strikingly, Skov-3 cells did not respond to cisplatin alone, but combined addition of cisplatin and GW reduced cell viability by 50 % (p<0.01). Furthermore, in combination with GI cisplatin reduced cell viability, but to a lesser extent (p<0.05) (Figure 3D). In summary, these data suggest that ADAM17 is essential for activation of survival pathways in cisplatin sensitive cell lines.

### Cisplatin-induced AREG release is selectively ADAM17 dependent

In order to assess the effect of ADAM17 activation on downstream survival signaling in ovarian cancer cells, we investigated activation of the EGFR pathway. Firstly, we measured the release of AREG as surrogate marker for ADAM17 activity (Figure 3A, 3B, 3C) and TNF-α receptor 1 (TNFR1), as another ADAM17 substrate (Supplementary Figure 2A). Secondly EGFR and ERK phosphorylation were determined (Figure 3D).

**Figure 4:**
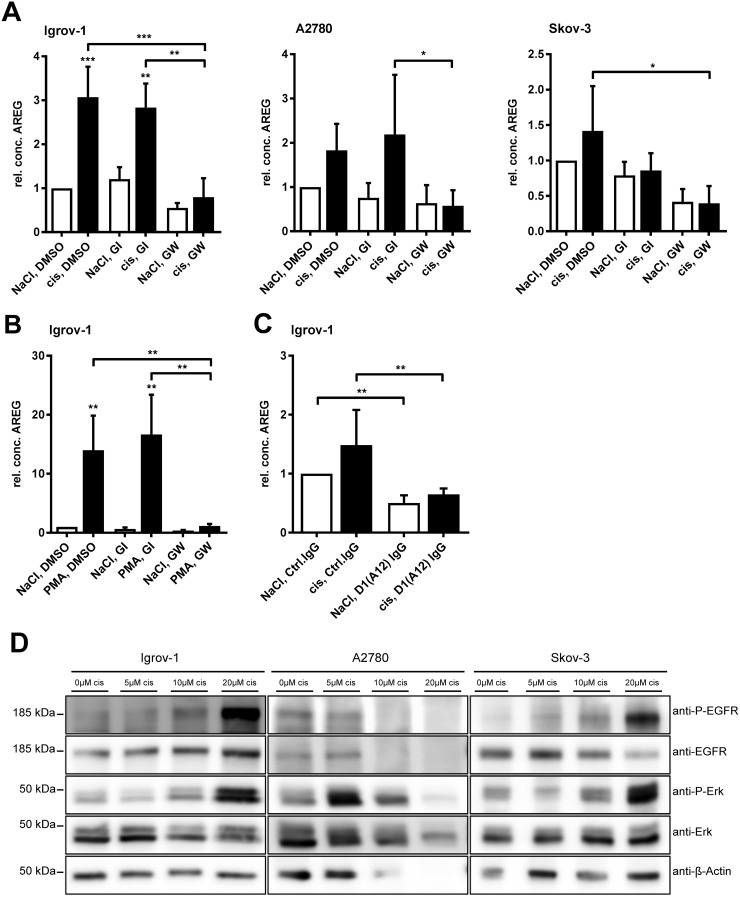
Cisplatin-induced AREG release is selectively ADAM17-dependent and cisplatin increases EGFR and ERK phosphorylation Cells were treated with 6 μM cisplatin or the equivalent volume of NaCl **(A)** 100 nM PMA (solved in DMSO) or DMSO **(B)** and 3 μM of the ADAM10 inhibitor GI or the combined ADAM10/ADAM17 inhibitor GW or DMSO for 24 h, as indicated (A, B). Additionally, ADAM17 was blocked by 200 nM of the anti-ADAM17 IgG antibody D1 (A12) or an equivalent amount of normal human IgG was used as a control **(C)**. AREG amounts in supernatants were investigated by AREG-ELISA and data were normalized to total protein amount of the cell lysates. For comparison of three independent experiments, the AREG-level of cells treated with NaCl and DMSO (A) or NaCl, Ctrl.IgG (C) was set to one. Means + SEM from three independent experiments are presented. Stars above black bars indicate differences between cisplatin treatment and the corresponding NaCl treatment (left). ANOVA; P = significance, ^*^ (p<0.05); ^**^ (p<0.01); ^***^ (p<0.001). For western blot analyses **(D)**, cells were treated for 48 h with the indicated amounts of cisplatin or NaCl as a solvent. Proteins were analyzed with the indicated primary antibodies. β-actin was used as a loading control. One of three representative blots is shown.

For direct comparison of apoptosis-induction (Figure 3B) and ADAM17 activity (Figure 4), we treated the cells in the same fashion as described above.

Already within 24 h of cisplatin treatment a significant release of AREG was detected in Igrov-1 cells (p<0.001). In all three cell lines tested, cisplatin-induced AREG release was inhibited by GW but not GI, indicating that AREG release was solely dependent on ADAM17 activity (Figure 4A). Similarly, TNFR1 cleavage was increased by cisplatin treatment of Igrov-1 and A2780 cells and reduced by inhibition of ADAM17 by GW, in contrast to inhibition of ADAM10 alone (Supplementary Figure 2A). Although no significant increase of AREG or TNFR1 was observed in Skov-3 cells upon cisplatin treatment, release of AREG and TNFR1 was decreased using the ADAM17 inhibitor GW.

For further confirmation of selective inhibition of ADAM17 by the inhibitor GW, we tested its inhibitory effect on

a) ADAM17 mediated AREG shedding induced by PMA and its subsequent inhibition by GW but not by GI (p<0.001) (Figure 4B).

b) functional inhibition of ADAM17 by the anti-ADAM17 antibody D1 (A12) [30], which decreased constitutive and cisplatin induced AREG-shedding, similar to GW (p<0.001) (Figure 4C).

In addition, siRNA mediated downregulation of ADAM17 revealed a significant reduction of AREG release in Igrov-1 cells (p<0.001) (Supplementary Figure 2B).

Taken together, cisplatin-induced AREG and TNFR1 release is mainly conducted by ADAM17 and not by ADAM10. This effect was most prominent in the cisplatin-sensitive, EGFR-expressing cell line Igrov-1.

### Cisplatin increases EGFR- and ERK phosphorylation

To investigate possible downstream mediators of ADAM17 activation, we analyzed phosphorylation of the EGFR and its downstream effector ERK, both playing a role in cell proliferation, viability and anti-apoptotic signaling [31, 32]. Igrov-1 and Skov-3 cells both responded dose-dependently to cisplatin with EGFR and ERK phosphorylation (Figure 4D). Moreover, A2780 cells showed an enhanced ERK activation upon cisplatin treatment, especially at initial doses of 5 μM and 10 μM.

Thus, cisplatin increased ADAM17 downstream effector activation in all three ovarian cancer cell lines.

### Cisplatin increases ADAM17 and AREG protein in primary ovarian cancer cells and triggers subsequent AREG release

Focusing the translational and clinical relevance of our research, we continued our investigations using ascites-derived (n=5) and tumor-derived (n=1) primary cells of patients, who were chemotherapy naïve.

**Figure 5:**
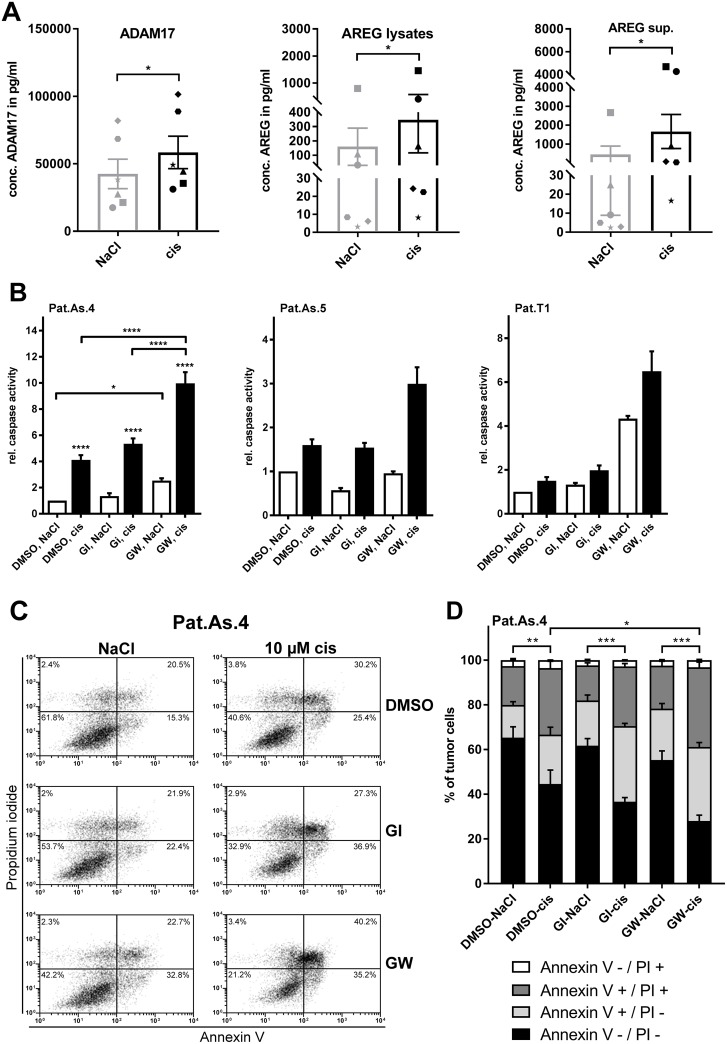
Blockage of ADAM17 effectively sensitizes patient-derived ovarian cancer cells to cisplatin Primary cancer cells were isolated from ascites of five ovarian cancer patients (Pat.As.1, 2, 3, 4, 5) and from ovarian cancer-tissue (Pat. T1). Patients are identified by different symbols. Following 6 days of cell treatment with 9 μM cisplatin or the equivalent amount of NaCl, cells were harvested. ADAM17 amounts in lysates and AREG levels in supernatants and lysates were determined by ELISA. The calculated concentrations were normalized to total cell number. Data are presented as mean + SEM of the patient collective **(A)**. For caspase and apoptosis measurement, cells were grown with and without addition of cisplatin [10 μM] or the equivalent amount of NaCl as a solvent for 48 h. To block ADAM10 and ADAM10/ADAM17, the inhibitors GI and GW were used at a concentration of 3 μM. Experiments were performed as three biological replicates for Pat.As.4 and are depicted as mean + SEM for this patient and mean + SD for Pat.As.5 and Pat.T1, where due to material restriction only technical replicates could be performed **(B)**. For FACS analyses 0.5×10^6^ Pat.As.4 cells were cultured for 48 h with NaCl (control) or with the indicated concentration of cisplatin (cis; diluted in NaCl) either in the presence of DMSO (control) or with GI or GW (each diluted in DMSO). After incubation, cells were washed and stained with Annexin V-FITC (An V) and PI. All analyses were measured on a FACS Calibur (BD Biosciences). **(C)** One representative experiment out of five independent ones is shown with the dot plot analysis. **(D)** The mean distribution + SEM of alive (An V/PI-negative), apoptotic (An V-positive, PI-negative), late apoptotic/necrotic (An V/PI-positive) or necrotic (An V-negative, PI-positive) Pat.As.4 cells of five independent experiments are presented. Stars above black bars indicate differences between cisplatin treatment and the corresponding NaCl treatment (left). Wilkox test (A) ANOVA (B, D); P = significance, ^*^ (p<0.05); ^**^ (p<0.01); ^***^ (p<0.001); ^****^ (p<0.0001).

Importantly, cisplatin treatment increased ADAM17 protein by about 20 % in all investigated primary cells (Figure 5A, left). Moreover, the protein concentrations of AREG were enhanced three to four times in these cells compared to non-treated cells (Figure 5A, central). Investigation of the corresponding supernatants collected six days after treatment showed a strong increase of AREG release in presence of cisplatin indicating a combinatorial effect of ADAM17 and AREG in these cells (Figure 5A, right). Likewise, we also observed a time-dependent increase of AREG in culture supernatants (data not shown).

### Blockage of ADAM17 sensitizes primary ovarian carcinoma cells to cisplatin

To validate the additive effect of ADAM17 blockage and cisplatin treatment in primary cells, we used patient-derived cells from tumor tissue (Pat.T1) and ascites-derived cells (Pat.As.4 and Pat.As.5), shown as examples due to restriction of primary material. For Pat.As.4 triplicates of caspase 3/7 activity assays and FACS analysis were performed.

Similar to Igrov-1 cells, cisplatin induced apoptosis in ascites-derived cells (Figure 5B). Additional blockage of ADAM17 doubled caspase activity in Pat.As.4 and Pat.As.5 cells (p<0.0001) compared to cisplatin only treatment. Interestingly, in Pat.As.4 addition of GW alone enhanced caspase activity, whereas in Pat.As.5 cisplatin was required to evolve increased apoptosis. Even in primary ovarian tumor-derived cells, where cisplatin alone was insufficient to induce caspase activation, selective inhibition of ADAM17 enhanced apoptosis four-fold. Combined treatment with cisplatin and GW increased caspase activity by six-fold (p<0.0001), indicating a strong additive effect of these therapeutics in patient-derived cells (Figure 5B).

FACS analysis of ascites cells (Pat.As.4) confirmed that the strongest induction of apoptosis was provoked by combined treatment with cisplatin and GW (p<0.05) compared to cells treated with cisplatin alone (DMSO, cis) (Figure 5C, 5D).

Taken together, inhibition of ADAM17 sensitized patient-derived ovarian cancer cells to cisplatin treatment.

## DISCUSSION

Chemo resistance is one of the major challenges in the treatment of ovarian cancer. Even though at least 70 percent of patients initially respond to platinum- and taxane based chemotherapy, the majority of advanced stage patients develop recurrent disease within the first 3 years after surgery and die within the first 5 years following diagnosis [33, 34]. Second and third line therapies with platinum backbone are re-initiated, if there was an initial response to platinum. However, response rates are commonly lower than those at initial diagnosis.

A major driver of chemo resistance is the enhanced activation of survival pathways such as EGFR, PI3K/AKT, MAPK signaling [8]. Recently, Carvalho et al. identified two important inducers of these pathways in advanced stage ovarian cancer patients undergoing chemotherapy [25]. These factors are the EGFR-ligands AREG and TGF-α, which both require proteolytic cleavage to be released from the surface membrane and become active mediators [18, 25].

With the aim to identify the upstream regulators of AREG and transforming growth factor-alpha (TGF-α) release, we focused our work on ADAM17, a metalloprotease, highly expressed in ovarian cancer and the major sheddase of these cell membrane hosted ligands [18, 35, 36].

In this study, we provide evidence that activity of ADAM17 and expression as well as shedding of the ADAM17 substrate AREG increase subsequently to chemotherapeutic treatment thus playing an important role in EGFR and ERK dependent chemo-sensitization of ovarian cancer cells.

Upon cisplatin treatment of the ovarian cancer cell lines Igrov-1 and A2780, we identified both, an enhanced AREG release as well as an increased AREG mRNA and protein expression in cell lysates. These results are in line with observations of Carvalho et al., who showed an enhanced activation of the AREG promotor following cisplatin treatment in the human ovarian cancer cell line MLS [25]. Moreover, we identified elevated AREG protein levels and a time-dependent AREG release in patient-derived primary ascites cells in *ex vivo* isolated primary cells (data not shown), thus extending the findings of Carvalho et al. by confirming a direct response mechanism of primary cells, isolated from chemotherapy naïve patients, which underscores the translational impact of our investigations. The fact that basal AREG-release of A2780 cells was significantly lower compared to Igrov-1 and Skov-3 cells indicates that this EGFR-deficient cell line is physiologically not dependent on AREG as an EGFR ligand.

Importantly, not only AREG levels were dramatically increased upon cisplatin treatment but protein amount of the metalloprotease ADAM17 itself also increased in the cisplatin-sensitive ovarian cancer cell lines Igrov-1 and A2780 as well as in ascites-derived primary cells. In contrast, the primarily cisplatin-resistant cell line Skov-3 did not respond to cisplatin with an increase of ADAM17 protein. Remarkably, the basal protein content of ADAM17 in Skov-3 was strongly enhanced compared to the other two cell lines. Thus, one potential mechanism of how Skov-3 cells develop chemo resistance may be due to an increase of ADAM17 protein, which constantly sheds its substrates thereby triggering survival pathways [35, 37]. As the basal AREG release of Skov-3 cells was similar to Igrov-1 cells, we conclude that other ligands than AREG are potentially more relevant to induce chemo resistance in Skov-3 cells.

However, no pronounced differences in mRNA content were detected between the different cell lines or upon cisplatin treatment supporting the notion that ADAM17 itself is mainly regulated post-translationally. Possible mechanisms of post-translational regulation were recently described by Dombernowsky et al., who showed that a sorting protein called PACS-2 (phosphofurin acidic cluster sorting protein) regulates recycling and stability of internalized ADAM17 and can divert ADAM17 away from degradative pathways [38].

The increased presence of ADAM17 is of critical relevance as it does not only shed AREG but was found to be the essential protease that releases five additional EGFR-ligands and a variety of factors which are crucial in tumor development and chemo resistance [18]. These include L1-CAM, Heparin-binding EGF-like growth factor (HB-EGF), TGF-α and heregulin, thus demonstrating the generalized relevance of ADAM17 promoting tumorigenesis [18, 29, 39–41].

Interestingly, it has been shown, that high expression levels of HB-EGF were significantly associated with the clinical outcome of ovarian cancer patients and correlated with ADAM17 expression [36]. Even though in the same publication no difference in survival was detected comparing patient cohorts, which revealed “low” vs. “high” ADAM17 expression, still an enhanced expression of ADAM17 was noted in ovarian tumors compared to normal ovaries. In contrast to this publication, Buchanan et al., observed a correlation of ADAM17 and progression free survival by using Affymetrix microarray data of grade 1 and 2 serous ovarian cancer patients [42]. These adverse outcomes may be due to different methods of patient selection: Whereas Tanaka et al. investigated data of grade 1, 2, 3 and 4, Buchanan et al. selected in their cohort for grade 1 and 2 patients [36, 42].

Even though these data seem to be controversial in the first place, both studies confirmed high expression of ADAM17 in ovarian cancer, but did not take into account that ADAM17 might be activated during chemo therapeutic treatment, as only patient specimens of chemotherapeutic naïve patients were investigated.

As ADAM17 activity is required for the release of HB-EGF into its soluble, active form, the publication of Tanaka et al. indirectly shows the relevance of ADAM17 presence in these tumors, as in absence of ADAM17 (if no other protease compensates for HB-EGF shedding), HB-EGF could not activate downstream signaling [18]. Here we aim to strengthen the point, that it is not only the presence, but even more the activity of ADAM17, being important for clinical outcome.

ADAM17 can be activated by a variety of factors, including G protein–coupled receptor (GPCR) agonists like ATP, lysophosphatidic acid (LPA) and thrombin as well as the protein kinase C (PKC) activator PMA (12-O-tetradecanoylphorbol-13-acetat) [10].

Depending on the ADAM17 activator, a distinct ADAM17 surface expression or internalization pattern was described. In line with our experiments, Lorenzen et al. showed a PMA-dependent short term increase of ADAM17 on the cell surface followed by rapid internalization, which was also demonstrated by Doedens and Black [43, 44]. As we observed a reduction of ADAM17 on cell surface following PMA and cisplatin treatment (48 h), we speculate that a similar mechanism of preceding ADAM17 activation might be the reason for this phenomenon.

Another important stimulator of ADAM17 is apoptosis: an increased activation of caspases was shown to enhance shedding of the ADAM17 substrate IL-6R [28]. A novel mechanism of ADAM17 activation was recently published by the group of Sommer/Reiss et al. [27], who demonstrated an enhanced ADAM17 activation triggered by surface exposure of phosphatidylserine during apoptosis. Considering this mechanism, the remarkably pronounced AREG-release upon treatment with high dosage of cisplatin may be explained by an increased apoptosis additionally to the augmented protein amounts of ADAM17 and AREG [27].

Measuring apoptosis, we demonstrated that ADAM17 blockage sensitizes ovarian cancer cells to chemotherapeutic treatment. Moreover, it may be considered that also ADAM10 or the combination of ADAM17 and ADAM10 inhibition play a role in apoptosis induction as also single treatment with the selective ADAM10 inhibitor GI led to a slight increase of apoptosis. This combinatory effect of ADAM17 or ADAM10 inhibition and cisplatin treatment has not been shown before in ovarian cancer, to the best of our knowledge. In line with our observations, Wang et al. demonstrated that overexpression of ADAM17 reduced cisplatin-induced apoptosis in HCC (hepatocellular carcinoma). Moreover, they showed that ADAM17-silencing sensitized cells to cisplatin under hypoxic conditions [31].

General tumor-suppressing effects of ADAM17 blockage have been demonstrated in previous studies [45]. For instance, functional inhibition of ADAM17 leads to decreased migration and proliferation in renal cell cancer [46, 47]. For ovarian cancer it has been demonstrated that an ADAM17-antibody reduces tumor growth in a xenograft model [30]; using the same antibody we confirmed the ADAM17 selectivity of AREG-shedding in our ovarian cancer cells. We confirmed that ADAM17 inhibition alone reduces cell viability in Igrov-1 and A2780 cells. In addition, combined treatment with cisplatin and ADAM17 inhibitor GW strongly reduced cell growth compared to treatment with only cisplatin, which highlights the potential role of ADAM17 inhibition to overcome resistance mechanism.

In conformity with Wang et al., who found increased EGFR and AKT activation in HCC cell lines, by forced ADAM17 expression [31], we demonstrated a cisplatin induced EGFR phosphorylation in Igrov-1 and Skov-3 cells. This activation process may be a consequence of increased ADAM17 and AREG expression and enhanced ADAM17 activity. In addition, we proved an activation of EGFR-downstream mediator ERK in all three ovarian cancer cell lines. These observations may indicate a direct impact of ADAM17 activation to inhibition of apoptosis and enhanced cell viability through activation of receptor tyrosine kinase (RTK) signaling.

Overall, this study provides evidence that ADAM17 crucially enhances chemotherapy induced cellular survival responses in cisplatin-sensitive cell lines Igrov-1 and A2780 as well as in patient-derived primary cells. In addition, basal protein content in the cisplatin-resistant cell line Skov-3 was strongly enhanced compared to sensitive cell lines indicating an intrinsic chemo resistance mechanism.

Thus, targeting ADAM17 in parallel to conventionally applied chemotherapy may represent a novel strategy to overcome resistance in ovarian cancer. Even though former approaches targeting ADAM17 was not successful in clinical trials due to the unspecific blockage of related metalloproteases, novel ADAM17-directed antibodies might be a promising tool for the treatment of ovarian cancer [19, 30, 48–50]. Recently Richards et al. successfully blocked ADAM17 in ovarian cancer, using an anti-human ADAM17 IgG antibody. They showed reduced tumor growth-rate and decreased shedding of the ADAM17 substrates TNFR1-α, AREG, and TGF-α in a xenograft model, upon ADAM17 inhibition [30].

Very recently, Sun et al. demonstrated that the treatment of cisplatin resistant ovarian cancer cells with the anti-PD-1 antibody Nivolumab led to downregulation of ADAM17 expression and increased apoptosis in these cell lines (Skov-3 and cisplatin resistant A2780 cells (A2780-DDP)) [51].

In addition, targeting of downstream mediators and receptors of ADAM17 signaling might enhance antitumor effects of chemotherapeutics, too. A neutralizing AREG antibody (AR30) e.g. revealed synergistic effects with cisplatin on the growth of human ovarian cancer xenografts [25].

Clarifying the contribution of particular growth factor receptors for the proposed signaling mechanism as we did so far for EGFR and ERK might enlarge the application spectrum of clinically approved inhibitors and antibodies to be considered for the use in a combinatorial setting. So far, most of these substances were restricted to second or third line treatment [52].

An example of these receptors could be the EGFR. We recently published that an EGFR-specific antibody theranostic conjugate could be used for highly specific detection and elimination of EGFR-positive cells [53]. This could be an effective first approach to limit the number of those cells, which most probably respond to the survival strategies induced by ADAM17 [53]. In conclusion this report highlights the relevance of ADAM17 and AREG in ovarian cancer, and possibly opens up new directions to overcome platinum resistance in ovarian cancer treatment.

## MATERIALS AND METHODS

### Ethics statement

This research was approved by the Institutional Review Board of the University Medical Center Schleswig–Holstein, Campus Kiel (AZ: B327/10) according to the Declaration of Helsinki. Written informed consent was obtained from all patients.

### Cell culture and isolation of primary cells

The human ovarian adeno-carcinoma cell lines Igrov-1 and Skov-3 were purchased from American Type Culture Collection (ATCC), the ovarian cancer cell line A2780 was obtained from SigmaAldrich (#93112519). Cells were cultured in RPMI-1640 medium including L-glutamine (SigmaAldrich, #R8758) with 10 % fetal bovine serum (Biochrom) and penicillin–streptomycin (pen.-str.) (30.000 U pen. / 30.000 μg str. per 500 ml RPMI-1640) (Biochrom). Cultivation was performed at 37 °C and 5 % CO_2_ in a humidified incubator. Cell lines were authenticated by Short tandem Repeat (STR) DNA profiling analysis before and during culturing as described previously [54] and routinely checked for mycoplasma contamination by MycoAlert™ (Lonza, #LT07).

Primary cells were isolated from advanced stage ovarian cancer patients during surgery at first diagnosis either from ascites or from tumor tissue. Ascites was centrifuged (348 g, 10 min), and the pellet was resolved in 12 ml RPMI-1640 medium, supplemented as described above and cells were seeded in a tissue culture flask, expanded and used for experiments when a confluency of ~75% was reached. Primary tumor cells were extracted from tumor tissue as described previously [55] and maintained in supplemented RPMI-medium, see above. We checked the polyploidic character of primary cells, using the tricolor probe TERC (3q26) / MYC (8q24) / SE 7 TC (Kreatech/Leica, #KBI-10704) for Fluorescence In Situ Hybridization (FISH). Cell fixation and FISH analysis were performed as described [56]. As polyploidy was confirmed in 60-100 % of each investigated patient sample, the majority of patient derived cells can be regarded as cancer cells.

### Chemotherapeutic treatment, cell lyses and ELISA

Cells were trypsinized and 1.5×10^6^ cells were seeded in 6-well plates. The next day cisplatin (obtained from the Clinic Pharmacy Services, UKSH, Campus Kiel at a concentration of 1 mM solved in NaCl) was diluted in NaCl for end concentrations of 5 μM, 9 μM, 10 μM and 20 μM. As a negative control the equivalent volume of NaCl was used (0 μM). Ovarian cancer cell lines were treated with these cisplatin dilutions for 48 h, primary cells were cultivated for six days. This timeframe was chosen for primary cells to investigate AREG release over time in a combined assay (see supplementary data). After the indicated cultivation time, supernatants were collected and centrifuged to save dissolved cells of the supernatants. Cells were trypsinized, resolved in medium and counted. Lysis of cells was performed as described previously [57]. Supernatants and lysates were measured using a human ADAM17 and a human Amphiregulin Duoset ELISA (R&D Systems, #DY930, #DY262) in NUNC-IMMUNO plates (Thermo Scientific, #442404), according to the instructions of the manufacturer. Optical density (OD) was measured at 450 nm with a microplate-reader (Infinite 200, Tecan). Concentrations were calculated using MS Excel (2010).

### Apoptosis assay

4×10^5^ cells were cultured in 12-well plates. The following day, cells were treated with 3 μM of the ADAM10 inhibitor GI254023X (Aobious, #3611) or the ADAM10/ADAM17 inhibitor GW280264X (Aobious, #3632). 120 min later, 6 μM or 10 μM cisplatin or the equivalent volume of the solvent NaCl was added to the culture medium. According to time-series experiments (data not shown), caspase activity of cell lines was measured after 24 h and caspase activity of patient-derived cells after 48 h of treatment. To do so, supernatants were centrifuged and the remaining cells of the pellet added to the trypsinized cells of the same well. These cells were centrifuged and re-diluted in 500 μl PBS. 25 μl of this cell suspension were transferred as triplicates into a 96-well plate (Corning Costar, #3917) to perform the Multiplex Assay ApoLive-Glo^TM^ (Promega, #G6411), which combines detection of viable cells and caspase-3/7 activity. This assay was conducted following the instructions of the manufacturer (TM325). Reagent volumes were adapted to the volume of the cell suspension. Cell viability values, measured as relative fluorescence units (RFU, 400_Ex_/505_Em_), were used to normalize the relative luminescence units (RLU) of the caspase-3/7 assay. Both assays were measured with a microplate-reader (Infinite 200, Tecan). Means were calculated with MS Excel (2010).

### Cell viability assay

To determine cell viability, 3-6×10^5^ cells were seeded in 6-well plates. The following day, cells were treated with 3 μM of metalloprotease inhibitors and cisplatin [6 μM] as described for the caspase assay. Viability was assessed after 48 h. To do so, cells were harvested as described above and measured by CellTiter-Fluor™ Cell Viability Assay (Promega, #G6080) as relative fluorescence units (RFU, 400_Ex_/505_Em_) with a microplate-reader (Infinite 200, Tecan). MS Excel (2010) was used to calculate means.

### Downregulation of ADAM17 by siRNA

1.5×10^5^ cells per well were seeded in serum and antibiotic free RPMI medium (24-well format). Cells were transfected according to Lipofectamine RNAiMAX reagent protocol (2013), using 10 pmol siRNA human ADAM17 (pool of three stealth RNAi: HSS110434, HSS110435, HSS186181; invitrogen/life technologies/Thermo scientific) or Control siRNA (ON-TARGETplus Control Pool, Dharmacon) and Lipofectamine® RNAiMAX transfection Reagent (Invitrogen, #13778) in two sequential transfection steps at day one and day two after seeding. 6 hours after the second transfection cells were incubated with 6 μM cisplatin or NaCl. Following 24 h at 37 °C supernatants were stored and cells harvested as described above. Supernatants were tested on AREG-levels, see below.

### ADAM17 activity measurement by AREG and TNFR1 ELISA

Cell lines were treated and harvested as described for apoptosis assay. Additionally, Igrov-1 cells were treated with either 100 nM PMA (Sigma-Aldrich) or 200 nM of the anti-ADAM17 IgG antibody D1(A12) [30] or the equivalent amount of normal human IgG Control (R&D Systems, #1-001-A).

To investigate ADAM17 activity, AREG-release into culture supernatants was measured by human Amphiregulin Duoset ELISA (R&D Systems, DY262) and human TNFR1 was quantified by Duoset ELISA (R&D Systems, DY225) by detecting the OD at 450 nm using a microplate-reader (Infinite 200, Tecan). ELISA results were normalized to the total protein amount of cell lysates, which were quantified by BioRad Dc Protein Assay (#500-0112), using MS Excel (2010).

### Westernblot and densitometry

Lysates were mixed in 5x denaturating buffer and boiled for 10 minutes at 95 °C. Proteins were separated by electrophoresis on 10 % SDS gels and transferred to PVDF membranes (GE health care). Membranes were incubated with the following primary antibodies in the indicated dilutions over night at 4 °C (anti-ADAM17 rabbit-Ab 1:2000 (Abcam, ab39162), anti-ADAM10 rabbit-Ab 1:1000 (Genetex, GTX63486), anti-Erk1/2 rabbit-Ab 1:1000 (Cell Signaling, #4696), anti-P-Erk1/2 mouse-Ab 1:1000 (Cell Signaling, #9101), anti-P-Histone rabbit-Ab 1:1000 (Cell Signaling, #2577), anti-EGFR rabbit-Ab 1:1000 (Cell Signaling, #6627), anti-P-EGFR rabbit-Ab 1:1000 Genetex (GTX61353), anti-β-Actin mouse-Ab 1:1000 (Sigma Aldrich, #A1978)). Horseradish peroxidase-conjugated secondary antibodies were incubated at room temperature for 1 h. For detection an ECL substrate kit was used (Thermo Scientific, St. Leon-Rot, Germany). Semiquantitative densitometry was performed using the Gels plugin in ImageJ. Intensities were normalized to β-actin.

### Flow cytometric analysis

3-5×10^5^ tumor cells (Igrov-1 or Pat.As.4) per well were treated with 6 μM or 10 μM cisplatin and 3 μM GI, GW the equivalent amount of DMSO or 100 nM PMA for 24 h or 48 h in 6-well plates. To study surface expression of ADAM17, cells were stained with 10 μl (pure) PE-labeled anti-ADAM 17 mAb (R&D Systems, #FAB9301P) and washed with wash buffer (PBS containing 1% BSA and 0.1% sodium azide). For analysis of cell death, cells were stained with Annexin-FITC following the procedures outlined by the manufacturer (MabTag, #AnxF100) except for a 1:50 dilution of Annexin V-FITC instead of a 1:20 dilution and with a final concentration of 1 μg/mL PI after a washing step with wash buffer. All samples were acquired on FACS Calibur flow cytometer (BD Biosciences) with FL2 channel for surface staining and FL1–FL3 channels for Annexin V-FITC and PI staining. Data analysis was done using the CellQuest Pro software (BD Biosciences).

### RNA isolation and real-time quantitative PCR (RT-qPCR)

Total RNA was isolated with total RNA kit peqGOLD (#12-6834-02; Thermo Fisher) and subjected to reversed transcription using the RevertAid First Strand cDNA Synthesis Kit (#K1621; Thermo Fisher) according to manufacturer’s instructions. The following primers were purchased from Sigma-Aldrich: hADAM17 (probe #78); 5‘ CAC CTTGCA GGA GTT GTC AGT 3‘; 5‘ CCT TCT GCG AGA GGG AAC 3‘ and 5‘ CAC CGA AAT ATT CTT GCT GAC A3‘; 5‘ CGG AGA ATG CAA ATA TAT AGA GCA C 3‘, PCR was performed as duplicate analysis with a LightCycler 480 (Roche) for maximum 50 cycles and melting curve analysis as quality control. The expression of genes of interest was normalized to gene expression of reference GAPDH.

### Statistical analysis

The data are represented as means + standard error. Data from at least three independent biological replicates, if not indicated differently, were used to test for normal distribution with the Shapiro-Wilk test using R (Bell Laboratories) or Graphpad Prism (GraphPad Software, Inc.). All following statistics were calculated using GraphPad Prism (GraphPad Software, Inc.). For parametric data, of matched datasets, one-way repeated measurement ANOVA was calculated for non-matched datasets ordinary one-way ANOVA was used, both were followed by Bonferroni correction for multiple comparisons. Non-parametric, matched datasets were analyzed by Friedman test followed by Dunn’s multiple comparison posthoc test. Non-parametric matched datasets of two treatment groups were analyzed with Wilkox matched-pairs signed rank test. P values < 0.05 were considered statistically significant.

## SUPPLEMENTARY MATERIALS FIGURES


