# Supplementary material for: ADAM17 inhibition enhances platinum efficiency in ovarian cancer
Source: Oncotarget. 2018 Mar 23;9(22):16043–58. doi: 10.18632/oncotarget.24682 (PMC5882316; doi:10.18632/oncotarget.24682)
Supplement: Supplementary file 1 [file oncotarget-09-16043-s001.pdf]

ADAM17 inhibition enhances platinum efficiency in ovarian cancer

SUPPLEMENTARY MATERIALS

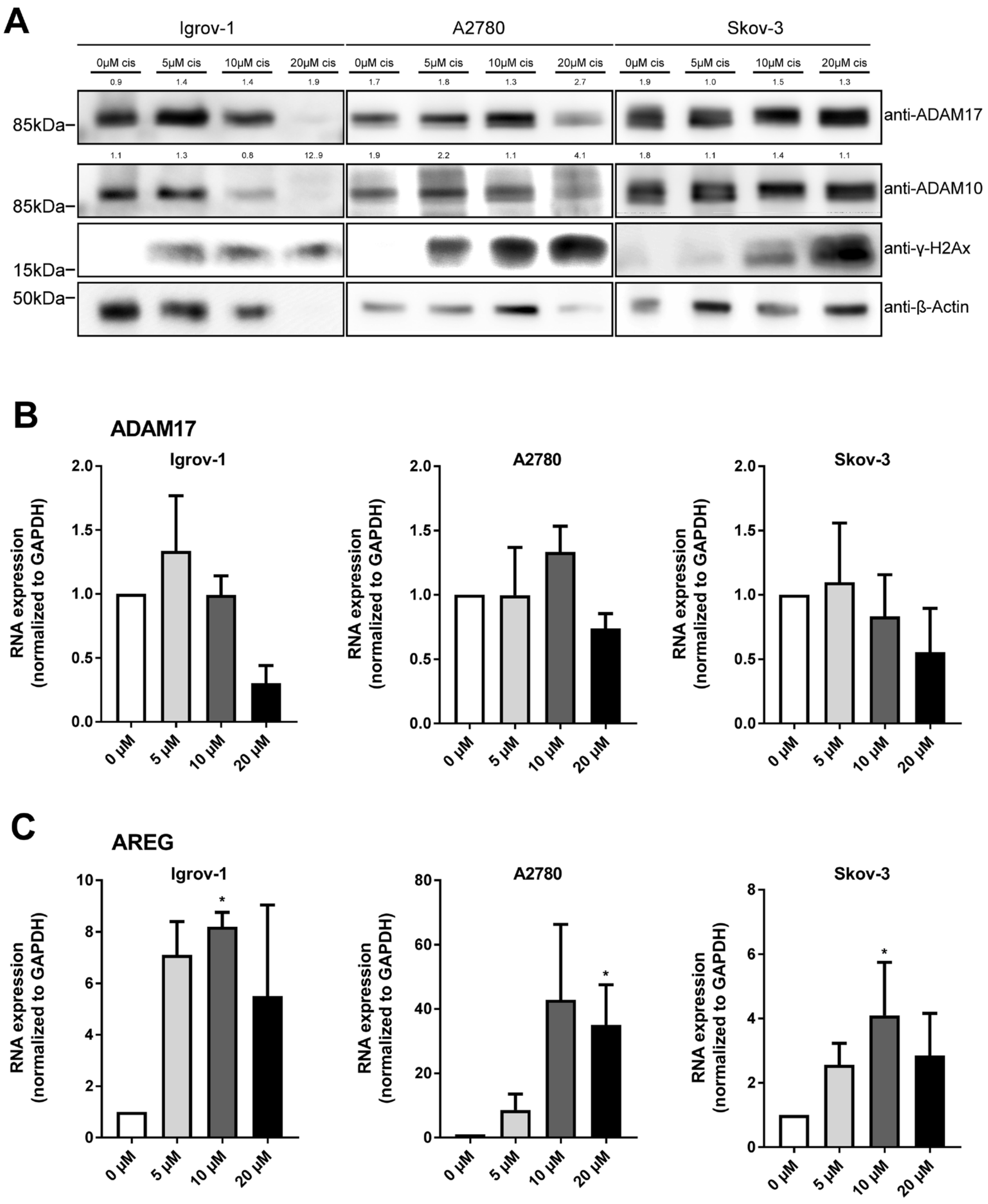

Supplementary Figure 1: Mature ADAM17 is expressed in ovarian cancer cells and cisplatin strongly increases AREG expression. Cells were treated with the indicated amounts of cisplatin or the solvent NaCl for 48 h. **(A)** For western blot analyses, cells were lysed and proteins separated by SDS page. Mature ADAM17 was expressed in all cell lines. Phosphorylation of the Histone H2AX (γH2x) indicates DNA damage and β-actin was used as a loading control. Note that with treatment of 20 μM cisplatin minor amounts of total protein are present due to increased cell death in cisplatin sensitive cell lines. Band intensities were quantified by densitometry using ImageJ. Intensities of ADAM17- and ADAM10-bands were normalized to intensities of the β-actin bands and are indicated above the respective lanes. One representative western blot out of three experiments is shown. **(B, C)** To determine mRNA expression, total RNA was extracted and after reversed transcription, PCR was performed and values of ADAM17 (B) and AREG (C) were normalized to GAPDH as a reference. Data of three independent experiments are presented as mean + SEM. ANOVA / Friedman test; P = significance, $^{*}p<0.05$.

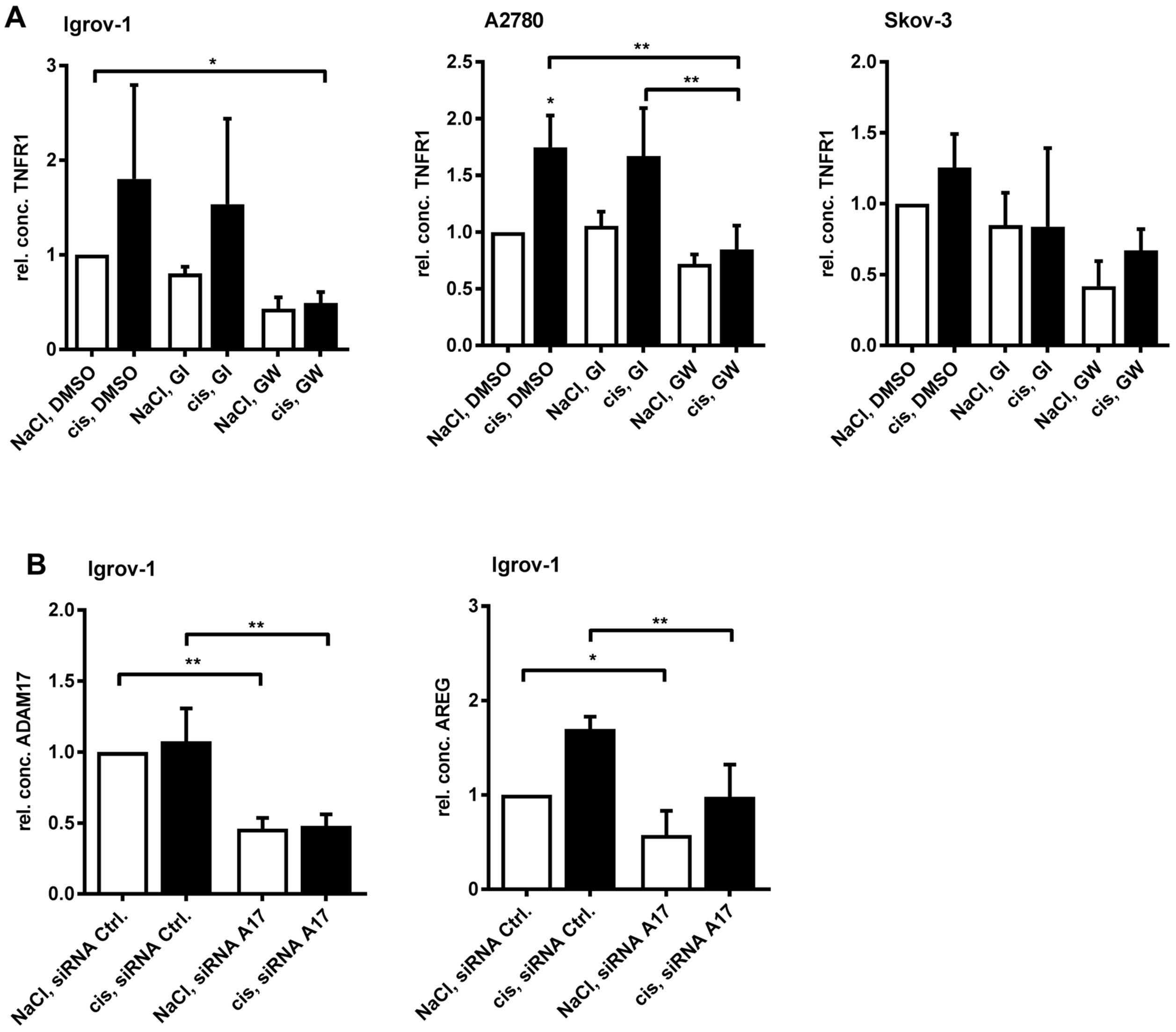

Supplementary Figure 2: Cisplatin induced TNFR1 and AREG-shedding is selectively ADAM17 dependent. (A) Cells were treated with 6 µM cisplatin or the equivalent amount of NaCl as a solvent either in the presence of 3 µM of GI or GW to block ADAM10 or ADAM10 and ADAM17, respectively, or DMSO which was used as a control solvent. After 48 h cells were harvested and supernatants investigated by TNFR1-ELISA. Cisplatin induced TNFR1 shedding, in Igrov-1 and A2780 cells, was blocked by GW but not by GI. **(B)** 1.5×10^5 cells were seeded in 24-well plates and transfected twice (once at day one and once at day two after seeding) with 10 pmol of ADAM17 siRNA or control siRNA. 6 h after the second transfection cisplatin or NaCl were added. After 24 h incubation, ADAM17 amounts in cell lysates and AREG-release into culture supernatants were investigated by sandwich ELISA. [Note that incubation time of cisplatin was only 24 h in contrast to Figure 1 (48 h), thus no difference in ADAM17 protein (NaCl vs. cis) is detected, yet.] ADAM17 protein levels were decreased by 50 % after transfection, irrespective of cisplatin treatment. Constitutive AREG shedding (white bars) and cisplatin induced AREG release (black bars) were both reduced by 50 %, when ADAM17 was downregulated. (A, B) Data of three independent experiments are presented as mean + SEM. ANOVA (B) / Friedman test (A); P = significance, $^{*}p<0.05$; $^{**}p<0.01$.
